# The physiological response of different tobacco varieties to chilling stress during the vigorous growing period

**DOI:** 10.1038/s41598-021-01703-7

**Published:** 2021-11-11

**Authors:** Kaiyuan Gu, Shuang Hou, Jinfen Chen, Jinge Guo, Fenfen Wang, Chenggang He, Congming Zou, Xiaoyu Xie

**Affiliations:** 1 College of Agronomy and Biotechnology, Southwest University, Chongqing, 400715 China; 2 Yunnan Academy of Tobacco Agricultural Sciences, Kunming, 650021 Yunnan China; 3 College of Tobacco Science, Yunnan Agricultural University, Kunming, 650201 China

**Keywords:** Plant sciences, Plant stress responses, Abiotic

## Abstract

Tobacco is be sensitively affected by chilling injury in the vigorous growth period, which can easily lead to tobacco leaf browning during flue-curing and quality loss, however, the physiological response of tobacco in the prosperous period under low temperature stress is unclear. The physiological response parameters of two tobacco varieties to low temperature stress were determined. The main results were as follows: ① For tobacco in the vigorous growing period subjected to low-temperature stress at 4–16 °C, the tissue structure of chloroplast changed and photosynthetic pigments significantly decreased compared with each control with the increase of intensity of low-temperature stress. ② For tobacco in the vigorous growing period at 10–16 °C, antioxidant capacity of the protective enzyme system, osmotic adjustment capacity of the osmotic adjusting system and polyphenol metabolism in plants gradually increased due to induction of low temperature with the increase of intensity of low-temperature stress. ③ Under low-temperature stress at 4 °C, the protective enzyme system, osmotic adjusting system and polyphenol metabolism of the plants played an insignificant role in stress tolerance, which cannot be constantly enhanced based on low-temperature resistance at 10 °C. This study confirmed that under the temperature stress of 10–16 °C, the self-regulation ability of tobacco will be enhanced with the deepening of low temperature stress, but there is a critical temperature between 4 and 10 °C. The self-regulation ability of plants under low temperature stress will be inhibited.

## Introduction

Temperature is an important factor affecting the distribution of plant growth area, as well as the main environmental factor affecting the growth and development of plants^1,2^. The appropriate temperature is one of the basic conditions for plant growth and development. When the plant's environmental temperature is lower than the specific temperature range at which plants grow, the minimum value, low intensity, scope and duration changes would lead to plant adaptive response. At the same time, the low temperature could make plant grow slowly, tissues and organs lose water and wilt, leaf turn yellow and show water stains, susceptible to diseases and insect pests, production decline in the quality and even plant die^3,4^. As a leaf cash crop widely cultivated, chilling injury is also a common natural disaster on tobacco production. According to the relevant research reports, in Yunnan, the largest growing area in China, low-temperature chilling injury causes serious damage to the tobacco growers. In the subsequent tobacco flue-curing process, it can also increases the brown reaction, which leads to a significant reduction in the apparent quality and taste, and causes huge economic losses^5,6^. Therefore, it has become the flash point and focus of tobacco abiotic stress research to study the physiological response mechanism of tobacco to low temperature stress, so as to reduce yield loss caused by cold injury.

At present, a large number of studies have been carried out around the world on the response of tobacco plant to low temperature stress. Zhou et al. studied the metabolism of polyphenols in tobacco seedlings under low temperature stress. It was found that low temperature stress could promote the synthesis of lignin in polyphenol metabolism of tobacco seedlings and enhance the protective effect of cell wall by increasing the content of lignin^7^. Li et al. treated K326 and HongDa in seedling growth stage at 4 °C for different stress time. The results showed that low temperature stress significantly inhibited the growth, photosynthesis and chlorophyll fluorescence characteristics of tobacco in seedling stage. HongDa had stronger low temperature tolerance than K326.Starting with a single variable of temperature^8^, Zhou et al. studied the effects of different low temperatures on the enzyme activities of two different tobacco varieties, and judged their tolerance to chilling injury^9^. Li et al. carried out field chilling injury experiments on mature tobacco. The results showed that low temperature stress could destroy the antioxidant enzyme system of mature tobacco leaves, reduce the quality of tobacco leaves, the content of key chemical components and production quality, and finally aggravate the occurrence of browning reaction of flue-cured tobacco^6^.

Although the physiological response of tobacco to cold stress has been explored in the above studies, it is not systematic enough, mainly focusing on seedling and mature stage of tobacco. The vigorous growing period is a very important period in the growth stage of tobacco. In recent years, tobacco chilling injury occurred frequently in the flourishing period in the tobacco growing area, especially in the high-altitude tobacco planting area, which is directly related to the formation of tobacco growth and yield in the later period. Therefore, it is of great practical significance to study the response mechanism of chilling injury in the vigorous growth period of plants, and there are rarely reports on the effects of different stress temperatures and different stress degrees on tobacco.

Flue-cured tobacco K326 and HongDa are the main varieties in Yunnan Province^10^. according to related research reports, there are great differences in tobacco root physiological dynamics characteristics and adaptability to different growth environment between the two varieties^11,12^. And in the long-term study, it was found that the two varieties showed different tolerance in low temperature environment. that is, K326 was a low temperature sensitive variety, while HongDa was a low temperature resistant variety^8,13–16^. Therefore, the selection of these two varieties not only has the practical significance of guiding agricultural production, but also can further understand the effects of chilling injury on tobacco varieties with different physiological characteristics.

Previous studies have shown that the physiological and biochemical responses of plants to low temperature are mainly manifested in four aspects: metabolic activity, cell membrane system, protective enzyme system and osmotic regulation substances^17^. Therefore, in this study, using two different varieties, three low temperature stress gradient experiments were carried out to determine the content of photosynthetic pigment, relative electrical conductivity (REC), antioxidant enzymes, osmotic regulation substances and polyphenol metabolites, and to observe the chloroplast by transmission electron microscope (TEM). On this basis, the physiological responses of two varieties to different degrees of low temperature stress were discussed, and the cold resistance of two tobacco varieties were compared. The purpose of this paper is to increase the understanding of the mechanism of tobacco response to chilling stress and to provide theoretical basis for practical production.

## Materials and methods

### Experimental materials

K326 and Hongda tobacco varieties provided by China Tobacco Seed Co., Ltd. were used in the experiment.

### Experimental treatment

The complete pelleted seeds of tobacco were selected and seeded in a seedling tray. The seedling substrate (Aihejia special substrate for floating seedling of flue-cured tobacco) was sterilized and deinsectized with carbendazim and trichlorfon in advance. The floating seedling was carried out in a greenhouse and managed by conventional seedling raising methods. When the seedlings grew to four leaves, the seedlings which grew well and had uniform size were selected and transplanted into a plastic basin (with a height of 17 cm and a diameter of 15 cm) containing seedling substrate. After transplantation, the tobacco seedlings were placed in an artificial climate incubator with the humidity of 75% at the temperature of 25 °C, under illumination of 20,000 LX for 14 h in the daytime and without light for 10 h at night. Half-strength Hoagland nutrient solution was applied once every three days at 9:00 a.m. till water was leaked from the bottom of the plastic basin. During this period, positions of the tobacco seedlings were randomly adjusted to ensure more consistent growth and illumination conditions for each tobacco. The biochemical analyses were conducted when the 11th leaf grew in this study.

### Experimental design

The tobacco plants with the same growth trend were selected and placed in four illuminated incubators. Each variety was divided into four groups, to separately carry out the following four treatments: treatment group at 25 °C (room temperature), treatment group under low-temperature stress at 16 °C (moderate), treatment group under low-temperature stress at 10 °C (severe) and treatment group under low-temperature stress at 4 °C (extremely severe). The experiment lasted for 6 days, and the fifth to seventh leaves from bottom up (with main veins and larger lateral veins removed) were collected for determining physiological indexes in each treatment group on days 0, 1, 2, 3, 4 and 5 under low-temperature stress. The samples were frozen in liquid nitrogen and preserved at − 80 °C. The samples for determining polyphenol content were taken (by green-killing of leaves at 105 °C for 15 min, drying and grinding leaves into powder at 75 °C, and sieving the powder using an 80-mesh sieve) and each sampling was repeated three times.

### Determination items and methods

#### TEM observation

The samples were cut into slices by an EM UC7 ultramicrotome (Leica, Germany) and stained with uranyl acetate and lead citrate. A HT7700 TEM (Hitachi, Japan) was used for observation and photography.

#### Determination of electrolyte leakage and malondialdehyde

The electrolyte leakage was determined by using the methods proposed by Chen et al. The contents of malondialdehyde (MDA) were determined by detection kits for MDA content (TBA colorimetry), visible light spectrophotometry) produced by Sino Best Biological Technology Co., Ltd., Shanghai, China.

#### Determination of chlorophyll content

The method proposed by Peng et al. was used, namely the mixture of acetone: ethanol = 2:1 was used to extract chlorophyll^18^.

#### Determination of protective enzyme activity

Preparation of crude enzyme solution: tobacco seedling leaves of 0.1 g were weighed and placed into a 2 mL EP tube with small steel balls, in which precooled phosphoric acid buffer solution of 1 mL was added (the pH values of the solutions for determining SOD, POD and CAT were 7.8, 6.0 and 7.5, respectively). The samples were rapidly ground at low temperature of 4 °C by utilizing a JXFSTPRP-48 sample-making machine produced by Shanghai Jingxin Industrial Development Co., Ltd. and the solution was centrifuged at 12,000 rpm for 10 min. After that, the supernatant, namely crude enzyme solution, was taken for determination. SOD, POD and CAT were separately determined by nitro-blue tetrazolium, guaiacol method and CAT detection kits (colorimetry) produced by Sino Best Biological Technology Co., Ltd.

#### Determination of content of osmotic adjusting substances

Soluble sugar (SS) and soluble protein (SP) contents were determined by SS kits and determination kits for protein contents (visible light spectrophotometry) produced from Sino Best Biological Technology Co., Ltd.

#### Determination of polyphenol oxidase, phenylalanine ammonia-lyase activity and total phenol content

They were determined by polyphenol oxidase (PPO) detection kits, phenylalanine ammonia-lyase (PAL) detection kits and detection kits for total phenol in plants (colorimetry) produced by Nanjing Jiancheng Bioengineering Institute, China, respectively.

### Statistical method for experiments

The test data were statistically analyzed by Microsoft Excel 2006 and SPSS18.0 software and Duncan’s multiple range test was carried out. The data in this study was presented as the mean of three replicates and the significant level was set at alpha level (P < 0.05). The physiological indexes were comprehensively analyzed by utilizing the membership function method. When physiological indexes are positively correlated with stress effects, the membership function is shown as follows:

$${\text{U}}_{{\text{x}}} = ({\text{U}}_{{\text{x}}} - {\text{ U}}_{{{\text{min}}}} )/ ({\text{U}}_{{{\text{max}}}} - {\text{ U}}_{{{\text{min}}}} )$$

If physiological indexes have a negative correlation with stress effects, the membership function is expressed as follows:

$${\text{U}}_{{\text{x}}} = { 1 } - ({\text{U}}_{{\text{x}}} - {\text{ U}}_{{{\text{min}}}} )/ ({\text{U}}_{{{\text{max}}}} - {\text{ U}}_{{{\text{min}}}} )$$

where, U_max_ and U_min_ indicate the maximum and minimum values of a series of indexes, respectively; U_x_ represents the measured values of corresponding series of indexes. Finally, the weighted averages of membership function values of each index are calculated.

### Experimental material statement

The use of plants parts in the present study complies with international guidelines.

## Results and analysis

### Changes of chloroplast ultrastructure under different low-temperature stresses

**Figure 1:**
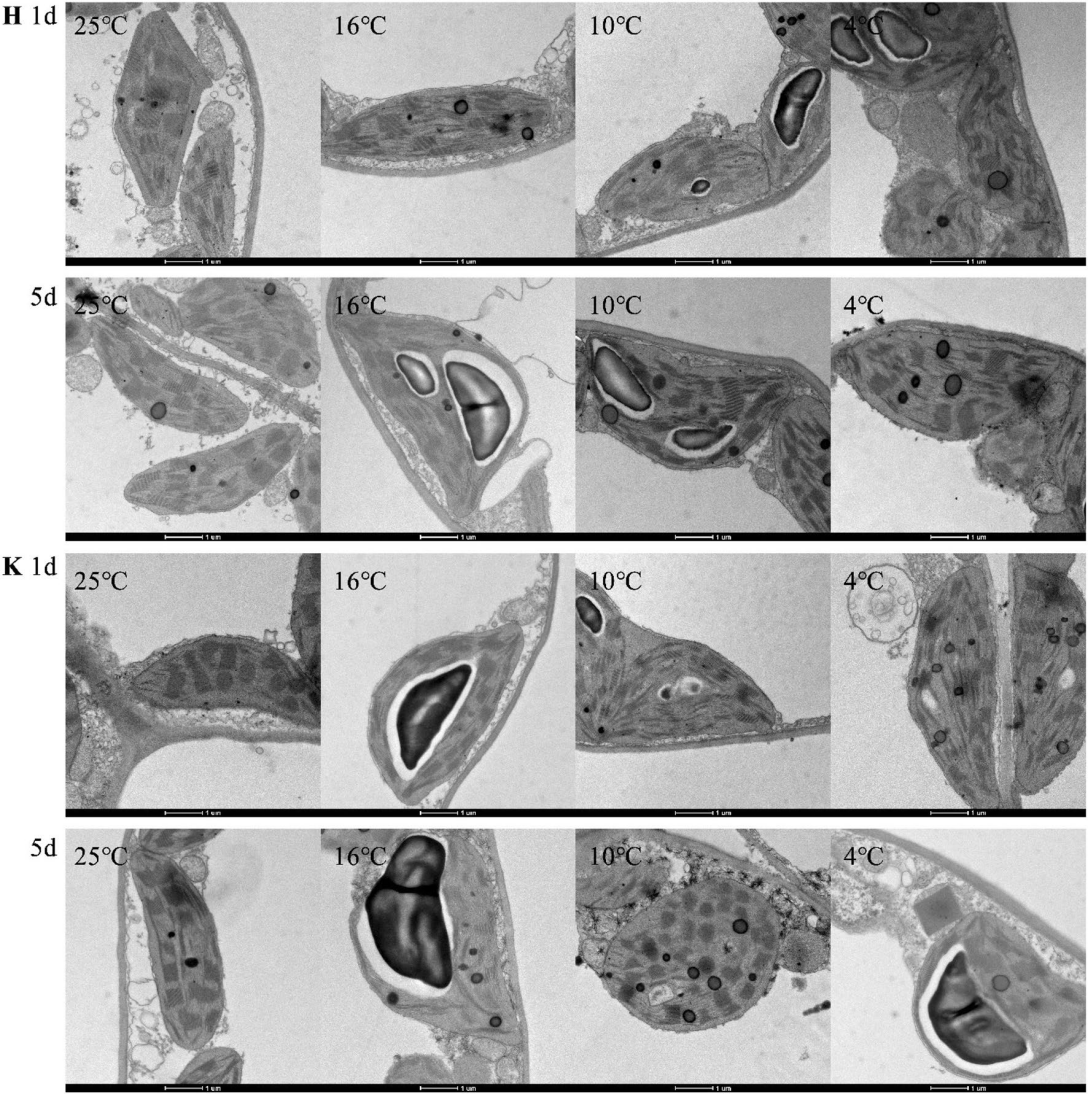
Effects of low-temperature stress on chloroplast ultrastructures in tobacco leave. "H" means Honghuadajinyuan variety, and "K" means K326 variety.

**Figure 2:**
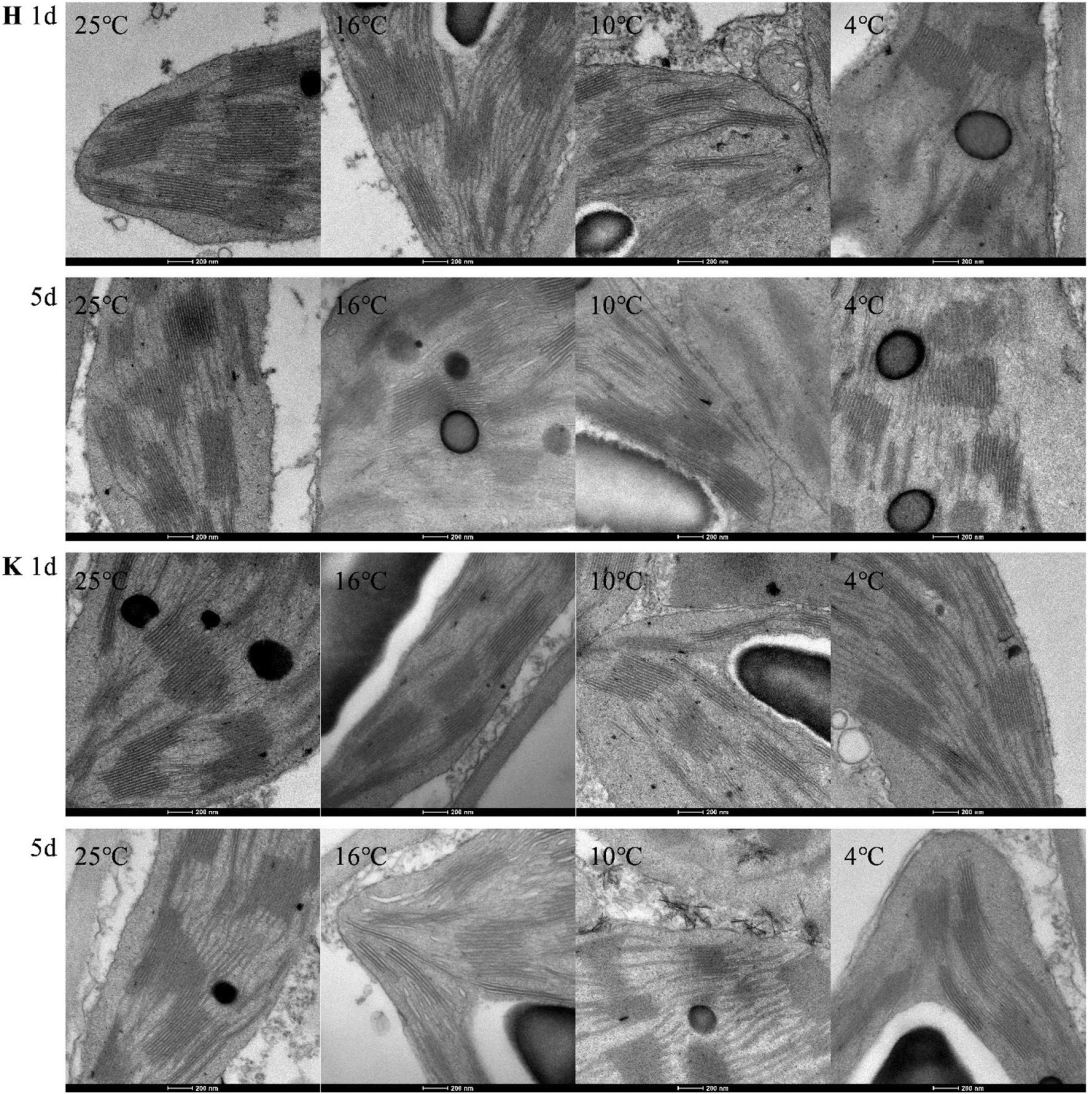
Effects of low-temperature stress on ultrastructures of grana lamellae of chloroplasts in tobacco leaves. "H" means Honghuadajinyuan variety, and "K" means K326 variety.

As shown in Figs. 1 and 2, at 25 °C, chloroplasts of tobacco leaves, which are oval or rhombic, were tightly attached onto the cell wall. The long axis was parallel to the cell wall, and starch granules were few and completely wrapped in chloroplasts. Moreover, grana lamellae were closely arranged, on which a small number of osmiophilic granules were attached. Compared with 25 °C, with increasing duration of low-temperature stress, the structure was destroyed and the membrane system was gradually broken. Starch granules in chloroplasts enlarged, and more osmiophilic granules appeared. Moreover, thylakoid lamellae became loose and distorted. For the same variety, the lower the temperature was, the more obviously the chloroplast structure changed. Under 16 °C treatment, Hongda variety with strong resistance on day 1 had no obvious difference with 25 °C. On day 5, the volume of starch granules significantly expended and a few osmiophilic granules appeared. Under 10 °C and 4 °C treatments, chloroplast structures in tobacco leaves changed obviously as early as day 1. On day 5, deformation degree of chloroplasts, volume of starch granules, degree of expansion and disintegration of thylakoids and number of osmiophilic granules reached the maximum. Under 16 °C treatment, K326 variety had a certain difference in chloroplast structure on day 1 compared with 25 °C, mainly shown as enlarged starch granules. On day 5 under 10 °C and 4 °C treatments, the volume of starch granules expanded obviously and a small number of osmiophilic granules were observed. Under 10 °C and 4 °C treatments, the change laws of chloroplast structures of K326 variety were same with those of Hongda variety. By comprehensively analyzing changes of chloroplast ultrastructures of the two tobacco varieties under low-temperature treatment, it is found that the chloroplast structure of K326 variety changed greater than that of Hongda variety under the same treatment.

### Influences of low-temperature stress on REC of tobacco leaves

**Figure 3:**
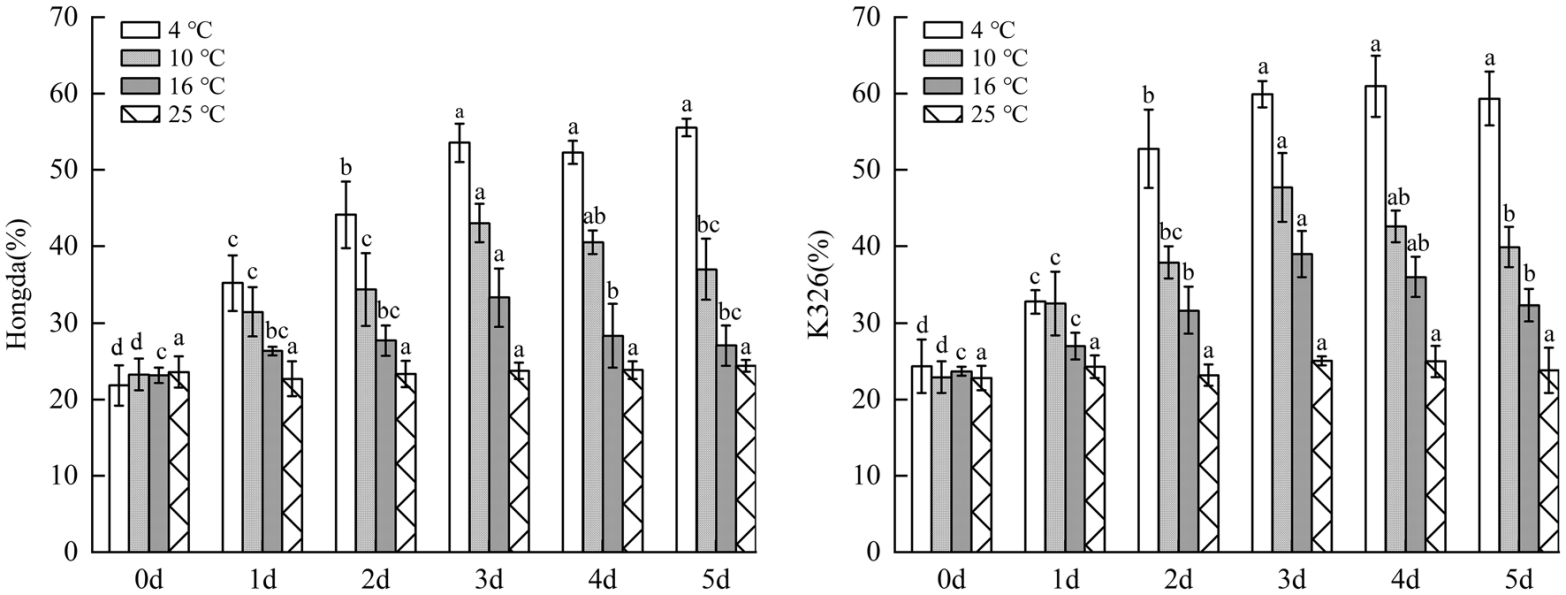
The changes of REC under different low-temperature stresses. Different lowercase letters indicated that there were significant differences at different times under the same temperature treatment (P < 0.05). The values were the mean of three biological replicates.

As demonstrated in Fig. 3, under 16 °C and 10 °C treatments, RECs of tobacco leaves of Hongda and K326 varieties firstly rose and then reduced with prolonging low-temperature stress, while they constantly increased with the increasing duration of exposure to stress under 4 °C treatment. Under the same low-temperature treatment, REC of leaves of K326 variety with weak low-temperature resistance rose largely compared with Hongda variety with strong low-temperature resistance. However, for the same variety, the lower the temperature, the faster the REC rose and the larger the increase amplitude was. REC of the low-temperature tolerant Hongda variety reached the peak on day 3 under 16 °C treatment, and then it reduced to the level before treatment (day 0) on day 5. Under 10 °C treatment, the maximum REC of the plants appeared on day 3 and then reduced. Under 4 °C treatment, REC showed no decrease trend over 5 d and reached the highest level on day 3 and then stabilized at this level. Compared with 25 °C, the REC of leaves in each period showed an upward trend.

### Effects of low-temperature stress on MDA content in tobacco leaves

**Figure 4:**
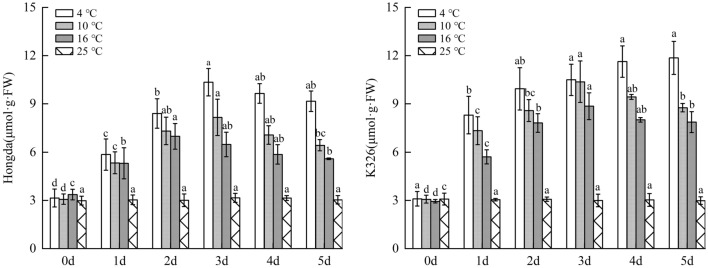
The changes of MDA content under different low-temperature stresses. Note: Different lowercase letters indicated that there were significant differences at different times under the same temperature treatment (P < 0.05). The values were the mean of three biological replicates.

As shown in Fig. 4, under 16 °C and 10 °C treatments, the MDA content in leaves of Hongda and K326 varieties firstly increased and then decreased, while they rose under 4 °C treatment with the increase of days of exposure to stress. Under the same low-temperature stress, MDA content in leaves of K326 variety with weak low-temperature resistance was larger than that of Hongda variety with strong low-temperature resistance in terms of increase amplitude. For the same variety, the lower the temperature, the faster the MDA content rose and the larger the increase amplitude was. MDA content in Hongda variety reached the peak on day 2 under 16 °C treatment and then decreased. Under 10 °C and 4 °C treatments, the contents reached the peak both on day 3. After that, the MDA content under 10 °C treatment decreased, while that under 4 °C treatment maintained at the peak. For K326 variety, the peak MDA contents were all found on day 3 under the three low-temperature treatments, and then MDA content decreased under 16 °C and 10 °C treatments, while it kept at the highest level under 4 °C treatment.

### Impacts of different low-temperature stresses on content of osmotic adjusting substances in tobacco leaves

#### Effects of different low-temperature stresses on SS content in tobacco leaves

**Figure 5:**
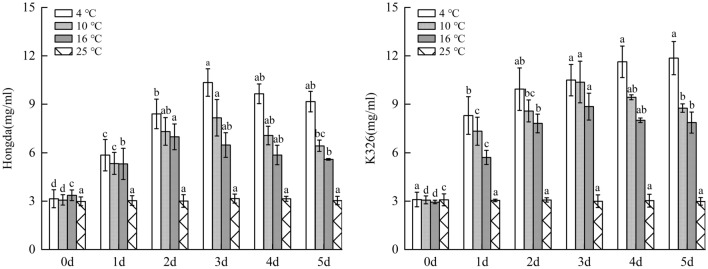
The changes of SS content under different low-temperature stresses. Different lowercase letters indicated that there were significant differences at different times under the same temperature treatment (P < 0.05). The values were the mean of three biological replicates.

As displayed in Fig. 5, SS content in tobacco leaves of Hongda variety under 16 °C, 10 °C and 4 °C treatments were significantly higher than those in 25 °C. With the increase of days under low-temperature stress, SS content under the three low-temperature treatments firstly increased and then decreased. The content reached the highest level under the three treatments on days 2 and 3, respectively. Under the three treatments, the content in leaves of K326 variety firstly rose and then reduced with the increase of days of exposure to stress and reached the highest on day 3. Under 10 °C treatment, SS content in leaves treated in 2–3 days continuously stabilized at the highest level and then reduced. The content of SS in leaves treated for 2 days under 4 °C treatment dramatically rose and reached the maximum on day 3.

#### Influences of low-temperature stress on SP content in tobacco leaves

**Figure 6:**
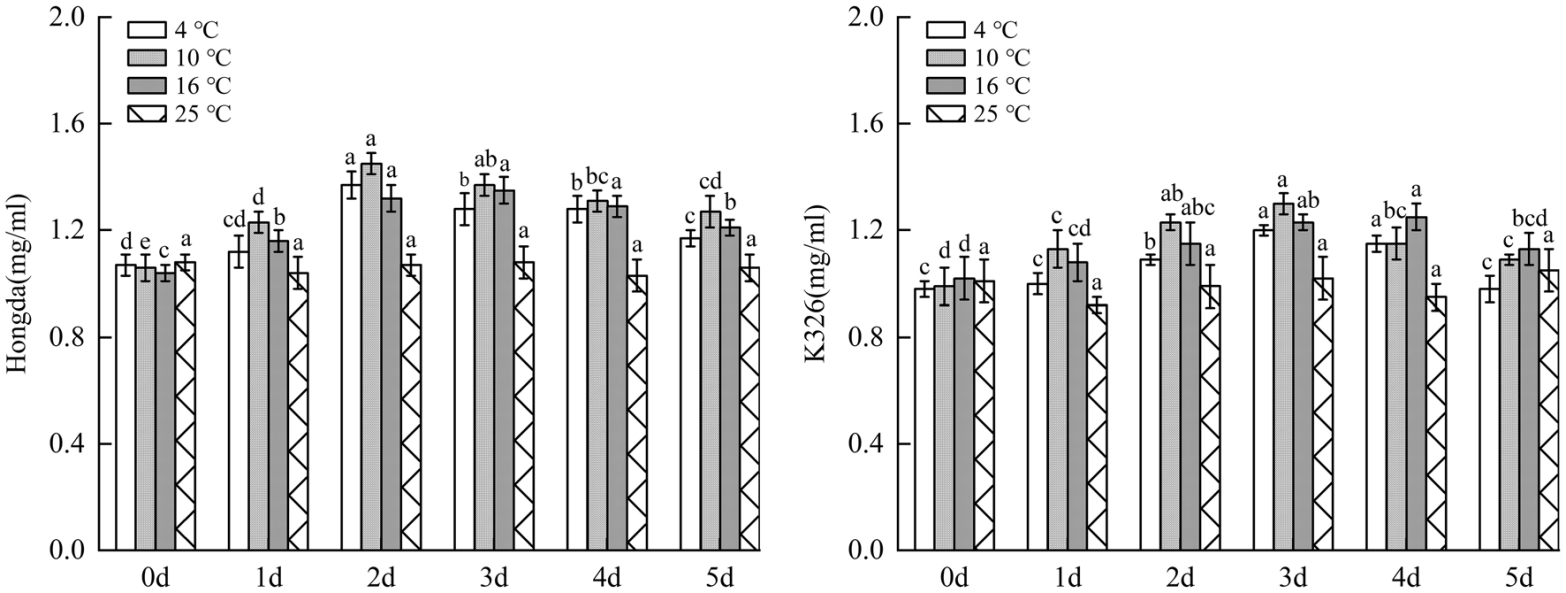
The changes of SP content under different low-temperature stresses. Different lowercase letters indicated that there were significant differences at different times under the same temperature treatment (P < 0.05). The values were the mean of three biological replicates.

Figure 6 illustrated that under 16 °C, 10 °C and 4 °C treatments, SP content in leaves of Hongda and K326 varieties both firstly rose and then reduced with the increase of days of exposure to low-temperature stress. Under the same low-temperature treatments, SP content in low-temperature resistant Hongda variety increased with a larger amplitude compared with the low-temperature susceptible K326 variety. For the same tobacco variety, SP content in plant leaves was the highest under 10 °C treatment, followed by that under 4 °C treatment, while the lowest content was found under 16 °C treatment. Under the three low-temperature treatments, SP content in Hongda showed a trend of first increased and then decreased with the rise of treatment days. Under 16 °C treatment, SP content in leaves maintained at the highest level in 2–4 days and then slowly decreased. Under 10 °C and 4 °C treatments, SP contents reached the maximum on day 2 and then begin to reduced. Moreover, the decrease rate of SP content under 4 °C treatment was faster than that under 10 °C treatment. Under the three treatments, SP contents in leaves of K326 variety firstly increased and then decreased with the increasing days of treatment. The highest SP contents were separately found on days 4, 3 and in 3–4 days and then reduced under these treatments.

### Changes of photosynthetic pigment in tobacco leaves under different low-temperature stresses

#### Changes of chlorophyll a content under different low-temperature stresses

**Figure 7:**
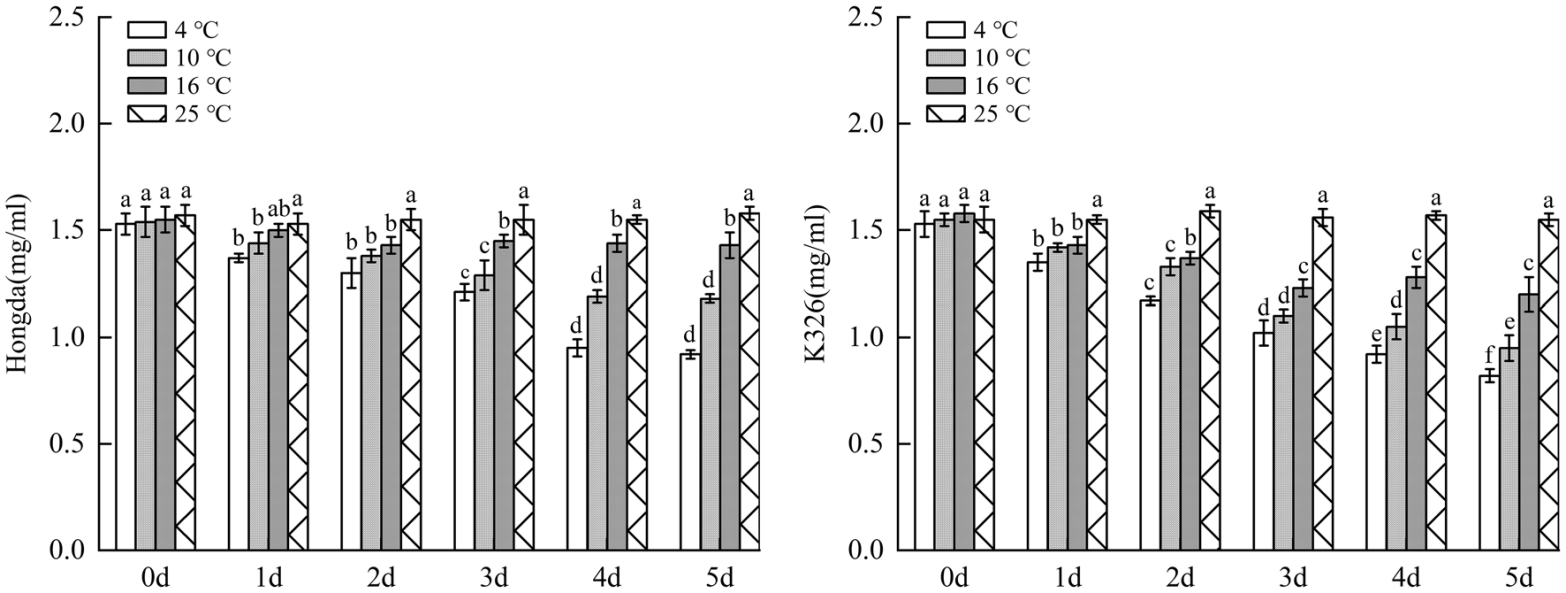
The changes of chlorophyll a content under different low-temperature stresses. Different lowercase letters indicated that there were significant differences at different times under the same temperature treatment (P < 0.05). The values were the mean of three biological replicates.

As demonstrated in Fig. 7, with the increase of days of exposure to low-temperature stress, chlorophyll a content in tobacco leaves decreased. Under the same low-temperature stress, the content of chlorophyll a in leaves of K326 variety with weak low-temperature resistance reduced faster than that in Hongda variety with strong low-temperature resistance. For the same variety, the lower the stress temperature, the faster the chlorophyll a content decreased. Under 16 °C treatment, chlorophyll a content in leaves of Hongda variety reduced to the lowest level as early as day 2. Under 10 °C and 4 °C treatments, chlorophyll a content on day 4 decreased faster and lasted for a longer time compared with that under 16 °C treatment. Under 16 °C treatment, chlorophyll a content in leaves of K326 variety reduced to the lowest level on day 3. However, under 10 °C and 4 °C treatments, chlorophyll a content constantly decreased over 5 days. The intensification of low-temperature stress rapidly and continuously reduced chlorophyll a in leaves of K326 variety.

#### Changes of chlorophyll b content under different low-temperature stresses

**Figure 8:**
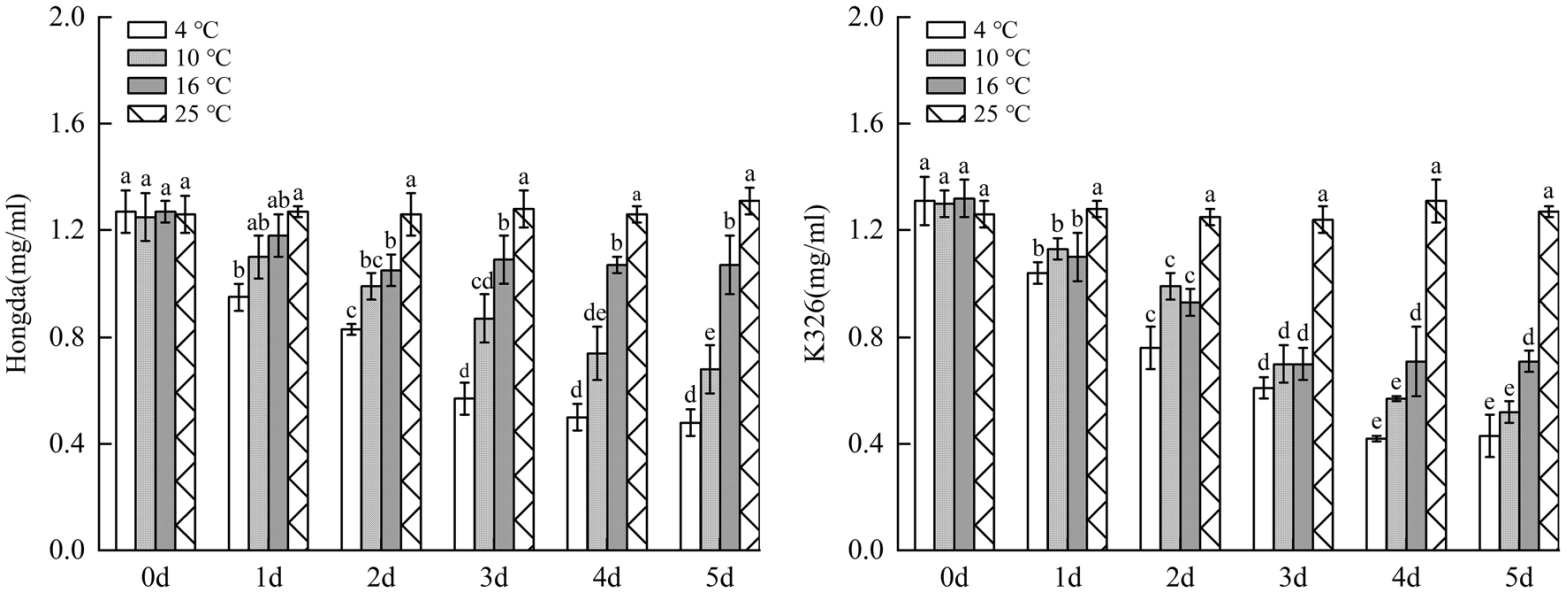
The changes of chlorophyll b content under different low-temperature stresses. Different lowercase letters indicated that there were significant differences at different times under the same temperature treatment (P < 0.05). The values were the mean of three biological replicates.

As displayed in Fig. 8, chlorophyll b content in leaves of Hongda and K326 varieties decreased with increasing days under low-temperature stress. Under the same low-temperature stress, chlorophyll b content in leaves of K326 variety with weak low-temperature resistance reduced faster compared with that of Hongda variety with strong low-temperature resistance. For the same variety, the more severe the low-temperature stress, the faster the chlorophyll b content decreased. Under 16 °C treatment, chlorophyll b content in leaves of Hongda variety reduced with a small amplitude and would not significantly decrease any longer from day 2. Under 10 °C treatment, chlorophyll b content constantly reduced over 5 days. In the early stage of 4 °C treatment, chlorophyll b content in leaves of Hongda variety dropped largely and from day 3, the content would not reduce and maintained at a very low level. For K326 variety, chlorophyll b content decreased slowly and would not reduce any longer from day 3 under 16 °C treatment. Under 10 °C and 4 °C treatments, chlorophyll b content stabilized at a low level in the later stage. During the reduction, the decrease amplitude of chlorophyll b content under 4 °C treatment was greater than that under 10 °C treatment.

#### Changes of chlorophyll a + b content under different low-temperature stresses

**Figure 9:**
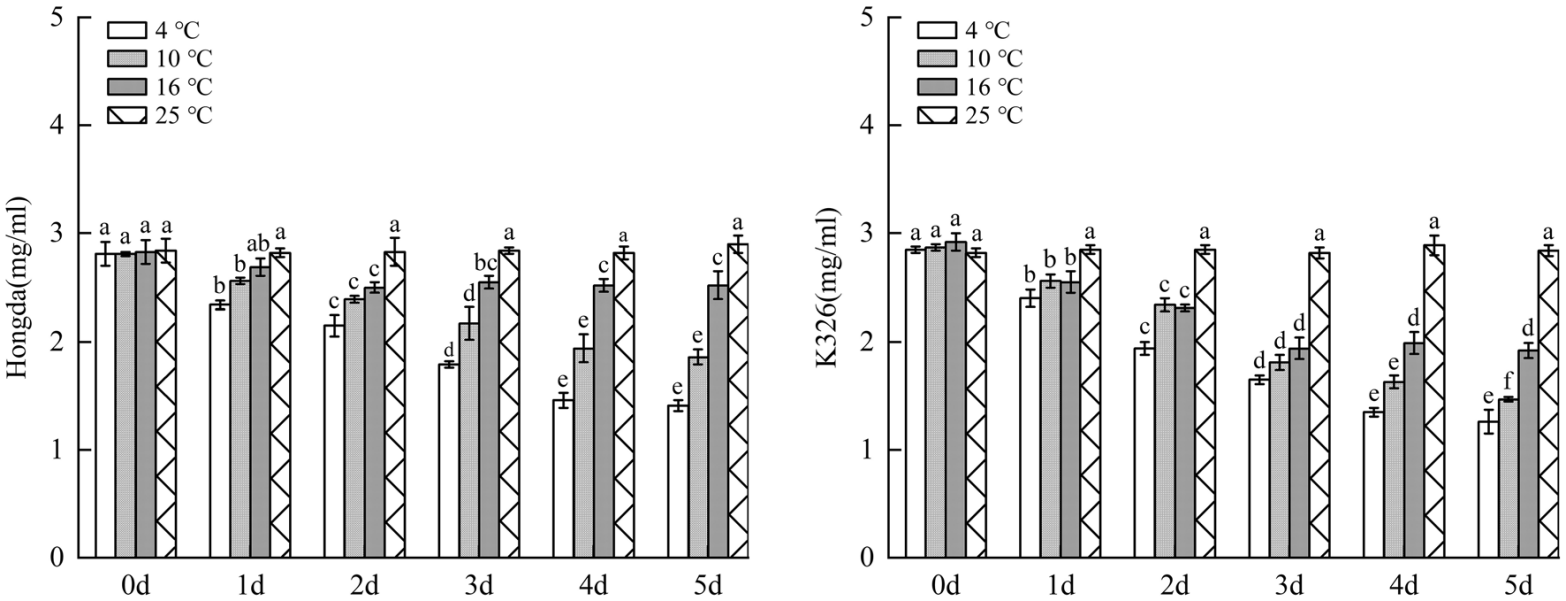
The changes of chlorophyll a + b content under different low-temperature stresses. Different lowercase letters indicated that there were significant differences at different times under the same temperature treatment (P < 0.05).The values were the mean of three biological replicates.

Figure 9 showed that chlorophyll a + b content in leaves of Hongda and K326 varieties both decreased with the increase of days of exposure to low-temperature stress under the three treatments. Under the same low-temperature stress, chlorophyll a + b content in leaves of K326 variety with weak low-temperature resistance reduced faster than that in Hongda with strong low-temperature resistance. As for the same variety, the lower the temperature, the faster the chlorophyll a + b content decreased. Under 16 °C treatment, chlorophyll a + b content in leaves of Hongda variety reduced with a small amplitude and there was no significant decrease trend in the content from day 2. Under 10 °C and 4 °C treatments, chlorophyll a + b content constantly dropped until the later stage and the decrease amplitudes were the largest under 4 °C treatment. Under 16 °C treatment, plants of K326 variety could rapidly inhibit decrease of total chlorophyll content. However, under 10 °C and 4 °C treatments, the total chlorophyll content continuously reduced with a large amplitude, which was unfavorable for plants to maintain normal photosynthesis.

### Effects of low-temperature stress on antioxidant enzyme system in tobacco leaves

#### Influences of different low-temperature stresses on SOD activity in tobacco leaves

**Figure 10:**
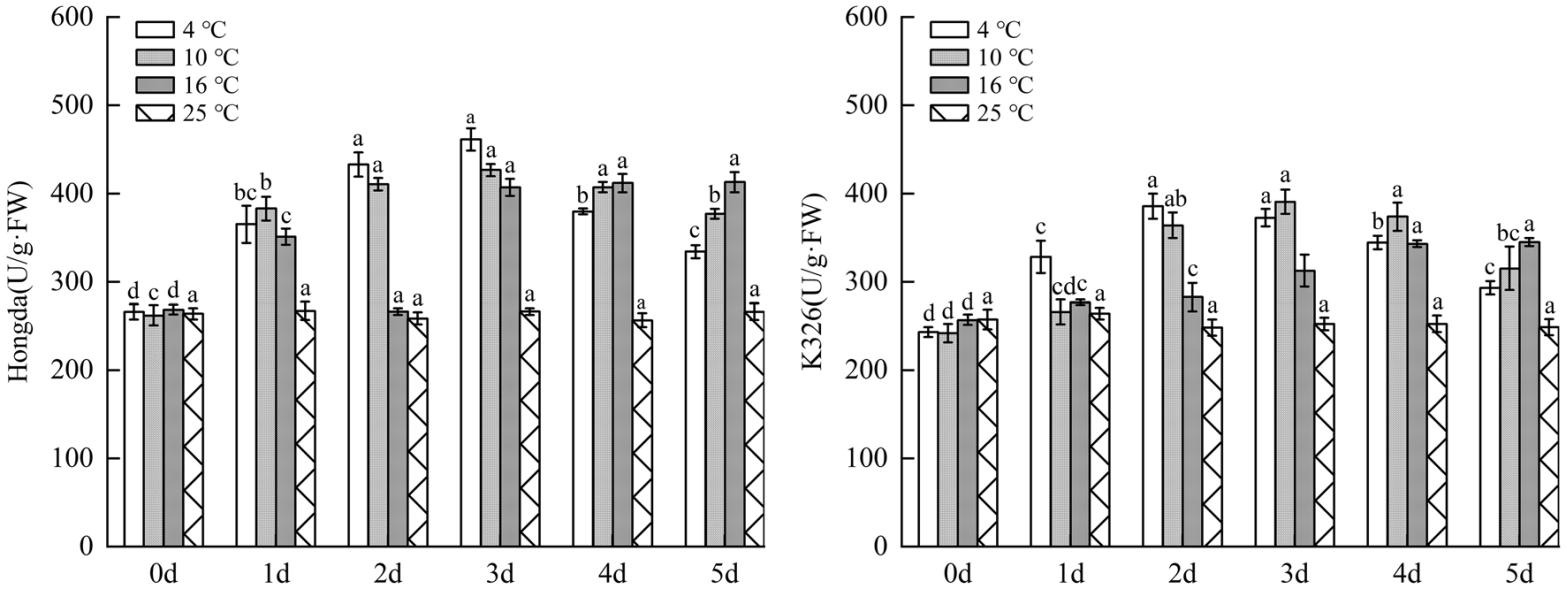
The changes of SOD activity in tobacco leaves under different low-temperature stresses. Different lowercase letters indicated that there were significant differences at different times under the same temperature treatment (P < 0.05).The values were the mean of three biological replicates.

As shown in Fig. 10, under 16 °C treatment, SOD activity in leaves of Hongda variety rose and tended to be stable with prolonging duration of low-temperature stress. SOD activity in 1–5 days significantly increased compared with 25 °C and reached the highest level in 3–5 days. Under 10 °C and 4 °C treatments, SOD activities in leaves firstly rose and then reduced with the increase of days under low-temperature stress based on the fact that they were remarkably higher than those in 25 °C. Under the two treatments, SOD activities separately reached the highest level in 2–4 days and 2–3 days and then began to decreased. For K326 variety with weak low-temperature resistance, the change trend of SOD activity in leaves was consistent with that of Hongda variety with the rise of days under 16 °C treatment. The activity in 1–5 days significantly increased compared with that in 25 °C and reached the highest level in 4–5 days. Under 10 °C and 4 °C treatments, SOD activities firstly rose and then reduced with the increase of days of exposure to low-temperature stress. The activities increased in comparison with 25 °C in each period, and they reached the maximum separately in 3–4 days and 2–3 days and then reduced.

#### Impacts of different low-temperature stresses on POD activity in tobacco leaves

**Figure 11:**
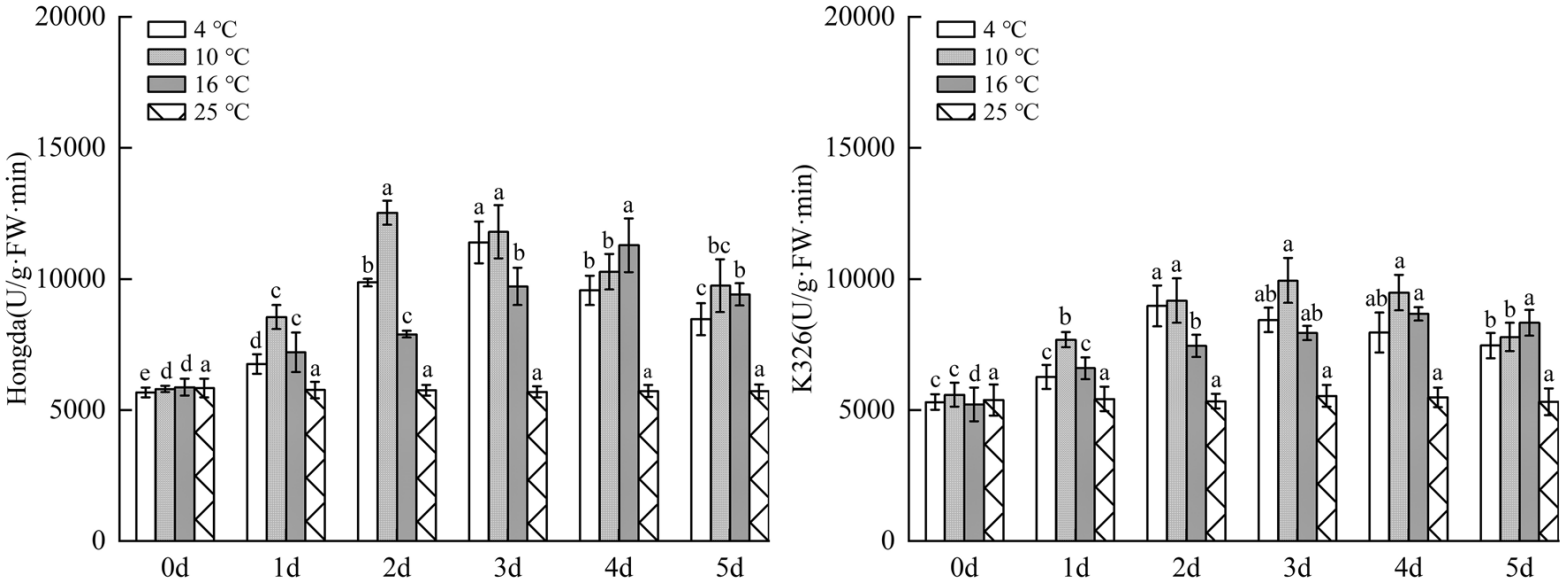
The changes of POD activity in tobacco leaves under different low-temperature stresses. Different lowercase letters indicated that there were significant differences at different times under the same temperature treatment (P < 0.05). The values were the mean of three biological replicates.

As demonstrated in Fig. 11, under 16 °C, 10 °C and 4 °C treatments, POD activities in leaves of Hongda variety significantly rose, and they firstly rose and then reduced with the increase of days under low-temperature stress. In 1–5 days, POD activities under the three treatments increased compared with those in 25 °C and reached the highest on day 4, in 2–3 days and on day 3, respectively. Under 16 °C treatment, POD activity in leaves of K326 variety firstly increased and then tended to be stable with prolonging days under stress and reached the maximum in 4–5 days. Under 10 °C and 4 °C treatments, POD activity showed a first increase and then decrease trend with the increasing days of exposure to stress. POD activities in each period under such treatments rose compared with 25 °C. The activity reached the highest in 2–4 days under 10 °C treatment and on day 2 under 4 °C treatment, respectively.

#### Effects of different low-temperature stresses on CAT activity in tobacco leaves

**Figure 12:**
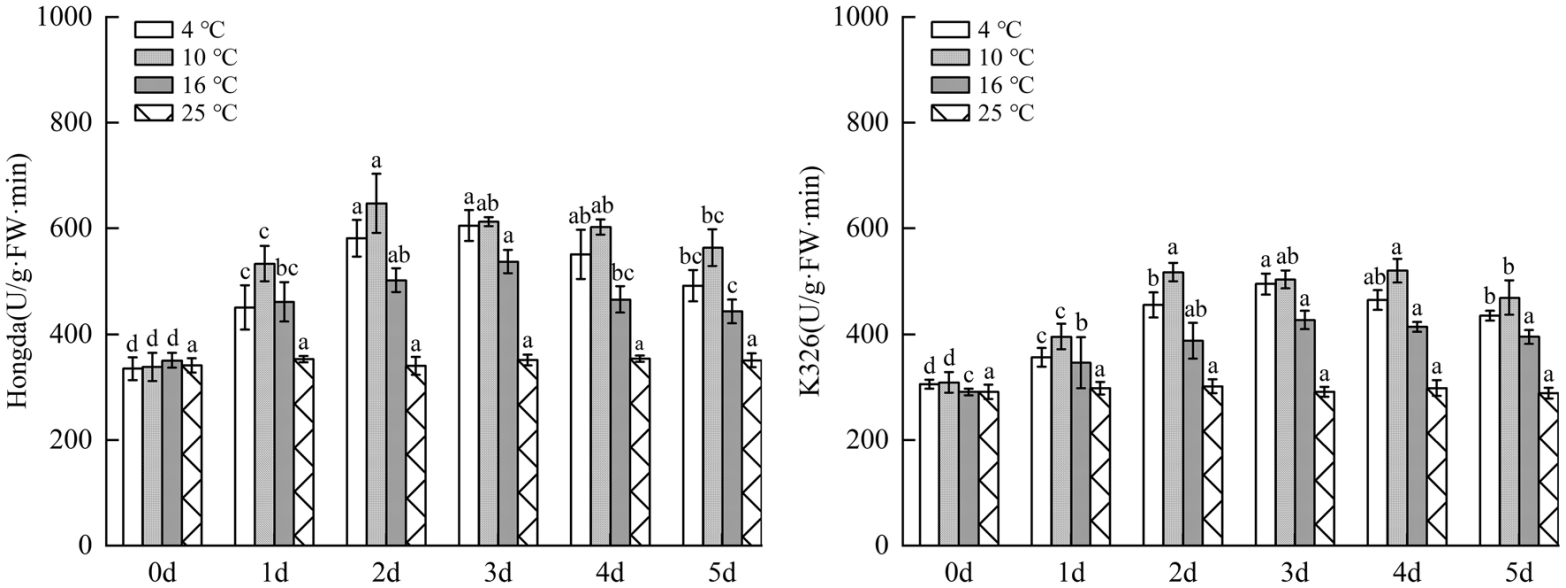
The changes of CAT activity in tobacco leaves under different low-temperature stresses. Different lowercase letters indicated that there were significant differences at different times under the same temperature treatment (P < 0.05).The values were the mean of three biological replicates.

As illustrated in Fig. 12, under 16 °C, 10 °C and 4 °C treatments, CAT activities in leaves of Hongda variety significantly rose due to low temperature and with the increase of low-temperature stress duration, they firstly rose and then declined on the whole. Under 16 °C treatment, CAT activities in each period increased compared with 25 °C and reached the maximum on day 3 and then began to reduce. Under 10 °C treatment, CAT activities in each period rose in comparison with 25 °C, reached the maximum on day 2 and then slowly decreased. Under 4 °C treatment, CAT activities rose in each period and maintained at the highest level in 2–3 days. CAT activity in leaves of K326 variety under 16 °C treatment notably increased compared with 25 °C. With the increase of days under stress, the CAT activity rose and then tended to be stable and kept at the highest level in 3–4 days all the time.

Under 10 °C and 4 °C treatments, CAT activities firstly rose and then reduced with the increase of days of exposure to stress on the basis of being significantly higher than 25 °C. By comparing with those in 25 °C in each period, the CAT activities were found to keep at the highest level in 2–4 days under 10 °C treatment and on day 3 under 4 °C treatment, respectively.

For the two tobacco varieties, low-temperature treatment could improve CAT activity in tobacco leaves. Under the same low-temperature treatments, the increase amplitude of CAT activity in leaves of the low-temperature resistant variety (Hongda) was greater than that of the low-temperature susceptible one (K326). For the same variety, the comparison of the maximum increase amplitudes of CAT activities under each treatment showed that the amplitudes were ranked in a descending order under 10 °C, 4 °C and 16 °C treatments. Moreover, the maximum CAT activity was inhibited under 4 °C treatment.

### Influences of low-temperature stress on polyphenol metabolism in tobacco leaves

#### Effects of low-temperature stress on PPO activity in tobacco leaves

**Figure 13:**
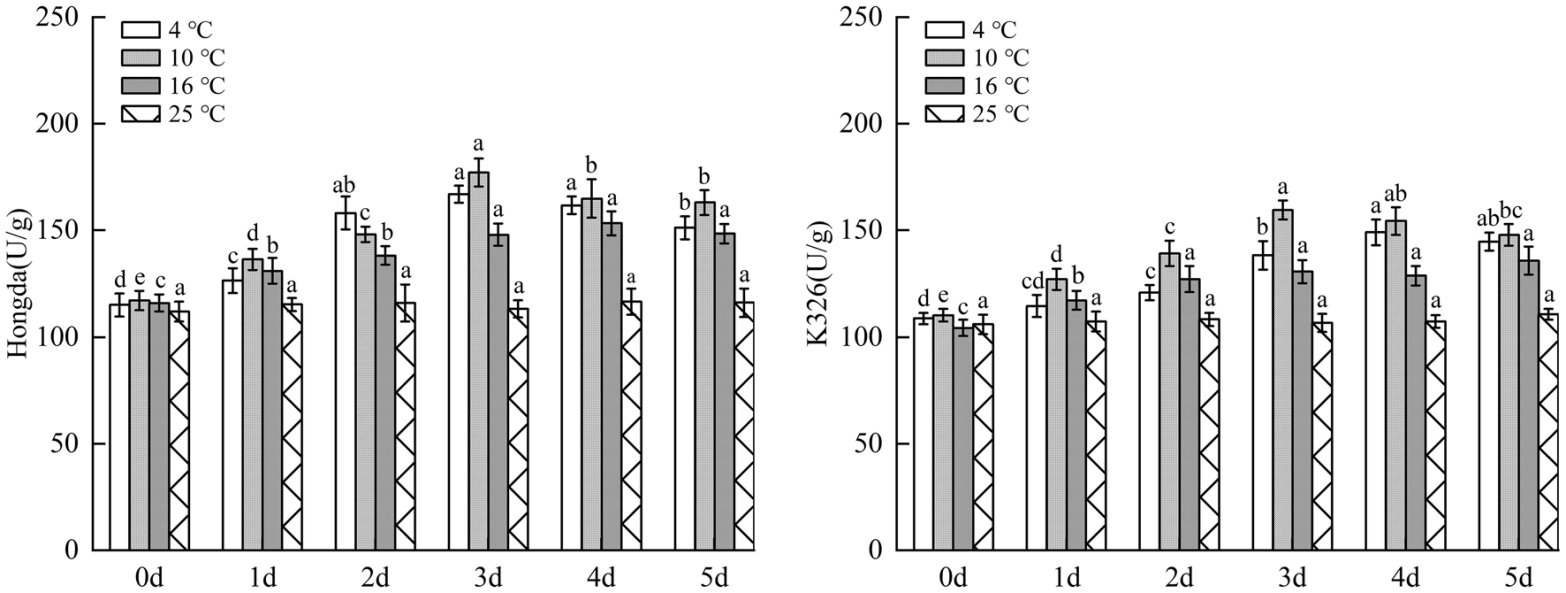
The changes of PPO activity in tobacco leaves under different low-temperature stresses. Different lowercase letters indicated that there were significant differences at different times under the same temperature treatment (P < 0.05).The values were the mean of three biological replicates.

Figure 13 illustrated that under 16 °C treatment, PPO activity in leaves of Hongda variety firstly increased and then tended to be stable with the increasing days under stress and reaches the maximum on day 3, while there was no significant difference in changes of PPO activity in 3–5 days. Under 10 °C and 4 °C treatments, PPO activities in leaves of Hongda variety were both significantly higher than those under 25 °C and they first increased and then decreased with the rise of days of exposure to stress. The activity reached the maximum on day 3 under 16 °C treatment and maintained at the highest level in 3–4 days under 4 °C treatment. Under 16 °C treatment, PPO activity in leaves of K326 variety showed the same change trend with Hongda variety, that is, it tended to be stable after rising. The activity reached the maximum after 2 d and then stabilized at this level. Under 10 °C treatment, PPO activity in leaves of K326 variety firstly increased and then decreased as stress duration prolongs. The activities in each period under the treatment rose compared with those in 25 °C and reached the maximum on day 3 and then reduced. Under 4 °C treatment, PPO activity in leaves of K326 variety slowly raised with time and reached the highest level on day 4.

#### Impacts of low-temperature stress on PAL activity in tobacco leaves

**Figure 14:**
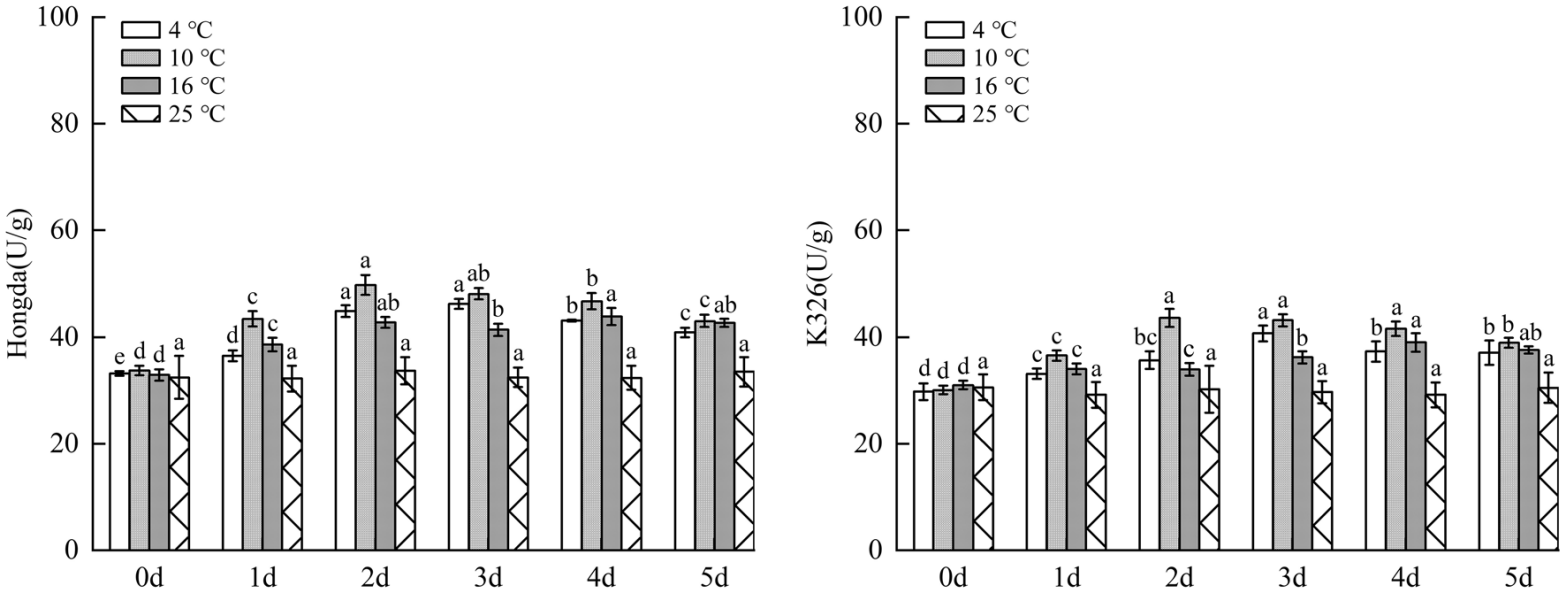
The changes of PAL activity in tobacco leaves under different low-temperature stresses. Different lowercase letters indicated that there were significant differences at different times under the same temperature treatment (P < 0.05). The values were the mean of three biological replicates.

As displayed in Fig. 14, under 16 °C treatment, PAL activity in leaves of Hongda variety significantly rose due to low temperature. With prolonging stress duration, the activity firstly increased and then tended to be stable; it reached the maximum on day 4 and then stabilized. Under 10 °C and 4 °C treatments, PPO activities in leaves notably increased at low temperature and they firstly rose and then declined with the increase of days under stress. PAL activities in each period under 10 °C treatment increased compared with those in 25 °C and reached the highest level on day 2. Under 4 °C treatment, the maximum PAL activity was found in 2–3 days. For K326 variety, PAL activity in leaves under 16 °C treatment significantly rose compared with 25 °C. With the increase of stress duration, the activity firstly increased and then tended to be stable, and it reached the highest level in 4–5 days. Under 10 °C and 4 °C treatments, the activities reduced after rising with prolonging stress duration. Under 10 °C treatment, the activities in each period increased in comparison with 25 °C and the highest PAL activity appeared in 2–4 days, while the maximum activity was found on day 3 under 4 °C treatment.

#### Influences of low-temperature stress on total phenol content in tobacco leaves

**Figure 15:**
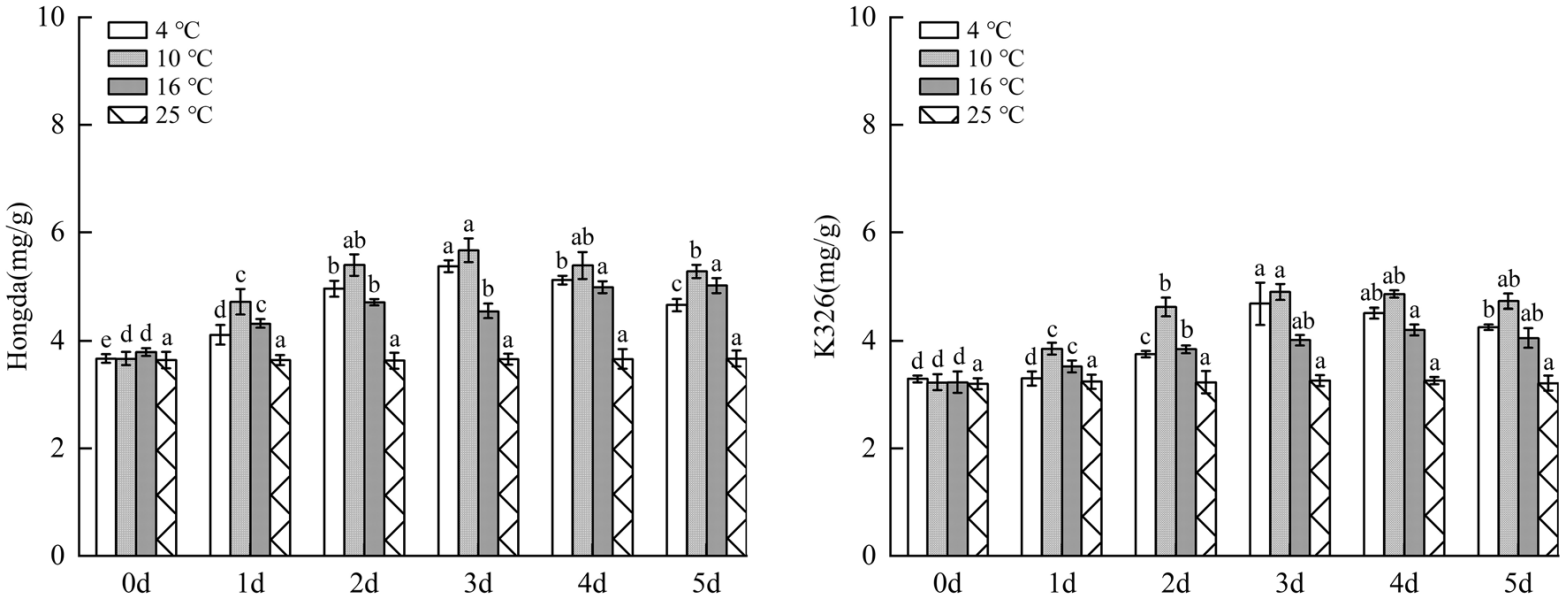
The changes of total phenol content under different low-temperature stresses. Different lowercase letters indicated that there were significant differences at different times under the same temperature treatment (P < 0.05).The values were the mean of three biological replicates.

As demonstrated in Fig. 15, under 16 °C treatment, as low-temperature stress duration prolongs, accumulation of phenolic substances in leaves of Hongda variety first rose and then is inclined to be stable, and the highest content was found in 4–5 days. Under 10 °C and 4 °C treatments, the content of phenolic substances firstly increased and then decreased with stress duration. In different periods, the content of phenolic substances rose compared with those in 25 °C and reached the highest level on day 3. In each period under 16 °C and 10 °C treatments, the content of phenolic substances accumulated in leaves of K326 variety increased compared with those in 25 °C and they rose and tended to stabilize with prolonging low-temperature stress duration. The highest contents under the two treatments were found on days 4 and 3, respectively. Under 4 °C treatment, the content of phenolic substances in leaves of K326 variety significantly increased compared with that on day 0. In addition, with the increase of days of exposure to stress, the content firstly rose and then reduced, and it reached the highest level on day 3 and then declined.

The results showed that for the same variety under different treatments, when the content of phenolic substances in leaves reached the highest level, the 10 °C, 4 °C and 16 °C were ranked in a descending order by the increase amplitudes of the content. In a certain temperature range, with the decrease of temperature, phenolic substances accumulated in leaves increased fast and their content rose. Under 4 °C treatment, as the strongest stress treatment in this experiment, the capacity of accumulating phenolic substances was lower than that under 10 °C treatment.

### Comprehensive analysis of membership function of cold resistance

As displayed in Table 1, through use of the membership function, a mathematical tool characterizing a fuzzy set, cold resistance of Hongda and K326 varieties were comprehensively evaluated based on ten measured indexes. By taking the average of membership degrees of the ten indexes as the comprehensive identification standard for cold resistance under such treatments, the larger the average was, the stronger the cold resistance, and vice versa. Data on day 5 under low-temperature stresses were selected for solving the membership function. The results showed that the average membership degrees of cold resistance indexes of Hongda variety under 16 °C, 10 °C and 4 °C treatments were 0.05, 0.83 and 0.12, respectively. Over 5 d of different low-temperature treatments, cold resistance of plants under 10 °C treatment was the strongest, followed by that under 16 °C treatment, while the weakest cold resistance under 4 °C treatment. The average membership degrees of cold resistance indexes of K326 variety under 16 °C, 10 °C and 4 °C treatments separately were 0.56, 0.75 and 0.16 and cold resistance under different low-temperature treatments were ranked in the same order with that of Hongda variety. The comprehensive results showed that tobacco still maintained under strong low-temperature resistance from 16 to 10 °C treatments for 5 days. The low-temperature resistance rose with the increase of intensity of low-temperature stress. Under aggravated stress, plants could improve their low-temperature resistance through self-regulation of metabolism, which was beneficial to reducing the damages of low temperature to plants. However, the weakest resistance of plants was shown under 4 °C treatment. In this case, the metabolism in plants may be seriously damaged by low temperature, and the reduction of low-temperature resistance would seriously damage plants under severe low-temperature stress.

**Table 1:** Membership function values and comprehensive evaluation of cold resistance indexes of two varieties under different low-temperature treatments.

Variety	Treatment (°C )	Stress duration/(d)											
		REC	MDA	SOD	POD	CAT	SS	SP	PPO	PAL	Total phenol	Average membership degree	Rank
Hongda	16	1	1	1	0.74	0	0	0.38	0	0.84	0.57	0.55	2
	10	0.18	0.62	0.54	1	1	1	1	1	1	1	0.83	1
	4	0	0	0	0	0.4	0.65	0	0.19	0	0	0.12	3
K326	16	1	1	1	1	0	0.37	1	0	0.28	0	0.56	2
	10	0.51	0.39	0.42	0.38	1	1	0.76	1	1	1	0.75	1
	4	0	0	0	0	0.54	0	0	0.73	0	0.29	0.16	3

## Discussion

### Changes of chloroplast ultrastructure under different low-temperature stresses

Chloroplasts are a kind of organelles which are susceptible to environmental changes. Their structural changes, the degree of thylakoid stacking, and the numbers of starch granules and osmiophilic granules change adaptively with the environment^19,20^. It is generally believed that Low temperature inhibits the synthesis and transportation of assimilation products. Products are rapidly transformed into starch and accumulated into large starch grains in chlorophylls^21^. The increase of starch grains is an adaptation of plants to low temperature environment, and osmiophilic granules are the result of degradation of thylakoid membrane of chloroplasts^22,23^. By observing tobacco seedlings with a SEM, Li et al. found that low-temperature stress can lead to deformation of chloroplasts, loose arrangement of grana lamellae, enlarging volume of starch granules and increasing number of osmiophilic granules, which is also confirmed in this study^8^. When the two tobacco varieties were treated at 16, 10 and 4 °C, osmiophilic granules appear and become increasingly more with prolonging stress time. Furthermore, the number of osmiophilic granules in K326 variety under each treatment was greater than that in Hongda variety, indicating that chloroplasts in tobacco leaves of the variety with good cold resistance confer better structural stability. Therefore, the adaptation of chloroplast structure to low temperature environment can be used as one of the criteria to judge the cold resistance of tobacco.

### Changes of photosynthetic pigment under different low-temperature stresses

Chloroplast pigment plays an important role in the growth and modulation of flue-cured tobacco^24,25^. In this experiment, chlorophyll a, chlorophyll b and total chlorophyll content in Hongda and K326 varieties under low-temperature treatments significantly reduced compared with those in 25 °C. With the increase of days of exposure to low-temperature stress, they constantly decreased with an increasingly large amplitude. For the same tobacco variety, chlorophyll a, chlorophyll b and total chlorophyll content under low-temperature treatments change consistently, that is, the lower the temperature is, the faster the decrease and the larger the decrease amplitude, which is consistent with the research results obtained by Liang et al.^26^. By comparing the two tobacco varieties under the same low-temperature treatments, it is found that chlorophyll a, chlorophyll b and total chlorophyll contentsin Hongda variety reduce slower with a smaller amplitude compared with those in K326 variety, which coincides with the research results of Chen et al.^27^. It also explain that the low temperature will break the balance of chlorophyll synthesis and degradation of the original, and result in changes of chlorophyll content. The strong low temperature resistant variety (HongDa) photosynthetic pigment drops less than the variety (K326) of which resistance is weak. On the one hand, it may be due to the weak chloroplast structure stability of the less resistant variety. At the same time, chloroplast synthase activity decreased significantly, and the low temperature caused the disorder of chloroplast function. On the other hand, it may be due to the oxidation rate of PSII electron transport primary quinone receptor QA is more restricted, and the photochemical quantum efficiency of PSII reaction center recovered more slowly^28^.

### Changes of REC and MDA content under different low-temperature stresses

REC is an index to measure the degree of electrolyte leakage in cells, and an important reference index for damage degree of plant cell membrane^29,30^. Qi et al. found that low-temperature stress can lead to disorder of plant cell membrane system and REC is positively correlated with the intensity of low-temperature stress, which is confirmed in this research^31^. Under the same treatment, the REC of K326 variety increased with larger amplitude than that of Hongda variety. RECs of the two varieties under the low-temperature treatment at 4 °C constantly increased, and when RECs reach the peak under each treatment, the increase amplitudes under 4 °C, 10 °C and 16 °C treatments were ranked in a descending order.

As one of the final products of membrane lipid peroxidation, MDA can also effectively reflect the damage degree of cell membrane^32,33^. The changes of MDA content in leaves of Hongda and K326 varieties under each treatment showed that for the same variety, the lower the temperature is, the higher the peak MDA content, which causes more serious damages to plants. Under the low-temperature stresses at 16 °C and 10 °C, plants can effectively reduce damages caused by low temperature in a short time through self-regulation of metabolism. However, under the low-temperature stress at 4 °C, the damages were not reduced in 5 days, which was consistent with the research results obtained by Wang Jiachuan concerning changes of relevant enzymes in different tobacco varieties induced by low-temperature^34^. This may be due to the damage of membrane lipids caused by reactive oxygen species under low temperature stress of 4 °C, which leads to the damage of cell function. It is consistent with the research results of Wang Jianchuan on the changes of related enzymes in different flue-cured tobacco varieties under low temperature induction. Under the same low temperature stress treatment, the increase of MDA content in leaves of K326 with weak low temperature resistance is greater than that of HongDa with strong low temperature resistance, which may be because the varieties with weak resistance are more sensitive to low temperature stress. Low temperature leads to the rapid increase of intracellular reactive oxygen species, which increases the degree of membrane lipid peroxidation and eventually produces a large amount of membrane peroxidation product MDA.

### Changes of content of osmotic adjusting substances under different low-temperature stresses

Zhang et al. found that SS and SP are important osmotic adjusting substances in plants and the increase of their content is positively correlated with cold resistance of plants^35^. The results showed that low-temperature stress rises SS content in tobacco leaves. Both Hongda and K326 varieties under 16 °C and 10 °C treatments showed that the capacity of accumulating SS in plants rose with the increase of intensity of low-temperature stress. However, under 4 °C treatment, the capacities of plants of the two tobacco varieties accumulating SS and SP were weakened compared with that under 10 °C treatment, especially K326 variety, with capacity to accumulate SS and SP even lower than that under 16 °C treatment. This indicates that under the treatment at 4 °C, the capacity of tobacco to enhance low-temperature resistance by regulating content of osmotic adjusting substances has decreased. At present, some reportse have pointed out that genes and functional proteins are involved in plant low temperature stress ^36^, but the research is not in-depth enough. In the future, we should explore the key genes that induce cold domestication and cold tolerance of tobacco^37^, especially the genes related to SS metabolism, and make use of modern biotechnology methods to improve the cold resistance of tobacco, so as to provide the basis for the breeding of good varieties and new varieties of tobacco germplasm resources with strong cold tolerance, and expand the cultivation range of tobacco.

### Changes of protective enzyme activity under different low-temperature stresses

SOD is one of the protective enzymes in plants^38^. It has been found that the resistance of plants is related to the maintenance of high SOD level in plants under stress^39^. The research results demonstrate that SOD activities of the two tobacco varieties both increase significantly under low-temperature treatment, and the increase amplitude of low-temperature tolerant variety is greater than that of low-temperature susceptible variety. Ma et al. also found that in a certain range of low-temperature intensity, SOD activity increases with the decrease of temperature^40^. However, when the temperature exceeds a certain critical value, its activity will decrease with intensifying stress, which is also confirmed in this study. Under 16 °C treatment, SOD activities in leaves of the two tobacco varieties both firstly rose and then tend to be steady. Under 10 °C and 4 °C treatments, SOD activities in leaves of the two varieties increased in the early stage and decreased in the later stage of low-temperature stress.

POD and CAT, as important enzymes to remove H_2_O_2_ in plants, play an important role in alleviating peroxidative damages and enhancing low-temperature resistance of plants under stress^41–43^. In this study, POD and CAT activities in leaves of the two tobacco varieties induced by low-temperature treatments both can significantly rise compared with those in 25 °C. Under 16 °C treatment, POD and CAT activities in leaves of K326 variety firstly increased and then tended to be stable with the increase of stress duration. Under 10 °C and 4 °C treatments, POD and CAT activities increased in the early stage and decreased in the later stage. For Hongda variety, under each low-temperature treatment, POD and CAT activities firstly rose and then declined with increasing stress duration, indicating that difference in cold resistance of the varieties was related to POD and CAT contents in leaves. For the same variety, the maximum increase amplitudes of POD and CAT activities were ranked in a descending order under 10 °C, 4 °C and 16 °C treatments. In other words, in a certain range of low-temperature stress, the lower temperature can stimulate higher POD and CAT activities in plants. However, when the temperature reaches 4 °C, the maximum enzyme activity is inhibited, which was consistent with the conclusion of Zhang et al.^44^. the reason may be in a certain range of chilling stress induced signal mechanism and cold stress response gene expression of tobacco, activate the protective enzyme system^45,46^, effectively tolerate freezing stress, while with the aggravation of stress, the production of H_2_O_2_ in plants increases, resulting in the decrease of POD and CAT scavenging efficiency. In the end, the damage to the plant caused by excessive H_2_O_2_ was aggravated.

### Changes of polyphenol metabolism under different low-temperature stresses

PPO and PAL are important enzymes in polyphenol metabolism of plants, which can effectively improve resistance of plants to diseases and insect pests and play an important role in resistance to external stress, and in photosynthesis and biosynthesis^47–49^. Under stress, high PPO and PAL activities are conducive to enhancing stress resistance of plants. This research showed that compared with 25 °C, the increase amplitudes of the highest levels of PPO and PAL activities in leaves of the same variety were ranked in a descending order under 10 °C, 4 °C and 16 °C treatments. Low temperature results in higher PPO and PAL activities. In addition, for different varieties under the same treatment, PPO and PAL activities in leaves of Hongda variety increased faster, with larger amplitude. This may be due to the activation of the defense system, especially the activation of phenylpropane metabolism, or the up-regulated expression of the upstream gene PAL, which promotes the increase of PAL activity to produce more plant protectants and lignin to alleviate the damage. These potential physiological responses can be used in the future molecular level study of tobacco cold resistance. Previous studies demonstrate that with the increase of intensity of low-temperature stress, PPO and PAL activities can be gradually enhanced^50^. However, in this experiment, the activities under 4 °C treatment were lower than those under 10 °C treatment, suggesting that extremely low temperature inhibits PPO and PAL activities to some extent.

Some studies showed that the more intensified low-temperature stress can stimulate plants to accumulate more phenolic substances to reduce damages of free radicals to plants^51^. This study illustrated that when the content of phenolic substances in leaves of the same variety under different treatments reached the highest level, the increase amplitudes were ranked in a descending order under 10 °C, 4 °C and 16 °C treatments. In a certain temperature range, with the decrease of temperature, the accumulation rate and content of phenolic substances in leaves increased. Under 4 °C treatment, as the strongest stress treatment in this experiment, the accumulation capacity of phenolic substances was lower than that under 10 °C treatment. The reason may be that under the treatment at 10–16 °C, tobacco plants were stimulated to accumulate a large amount of phenolic substances to reduce the damage of free radicals. Under the treatment at 4 °C, plants suffered from high-intensity stress, while their capacity to accumulate phenolic substances reduces. The pathway of removing lots of ROS produced under stress by using phenolic substances as non-enzymatic antioxidants in plants is blocked, so the low-temperature resistance of plants is relatively reduced.

## Conclusions

The change laws of Hongda and K326 varieties under the three low-temperature stresses were comprehensively analyzed. Based on this, it is found that for tobacco in the vigorous growing period under low-temperature stress at 10–16 °C, with increasing intensity of stress, antioxidant capacity of protective enzyme system, osmotic adjustment capacity of osmotic adjusting system and polyphenol metabolism are all enhanced by low-temperature stress. Such changes are conducive to timely improving the low-temperature resistance of plants, in order to survive under low-temperature stress. When temperature decreases to 4 °C, the protective enzyme system, osmotic adjusting system and polyphenol metabolism of plants fail to make a full function in stress resistance, which cannot be continuously enhanced on the basis of resistance at 10 °C, while instead inhibited to some extent. Two tobacco varieties, especially K326, are damaged seriously when they are exposed to 4 °C for a long time. The results of changes of chlorophyll, REC and MDA content and membership function of cold resistance indexes under different treatments prove the above conclusions. Therefore, it is considered that there is a critical temperature between 4 and 10 °C for tobacco in the vigorous growing period. When the temperature was lower than the critical value, the self-regulation capacity of tobacco plants under low-temperature stress begins to be inhibited.

## Data Availability

The data presented in this study is contained within the article.
